# Experiments, Which Tend to Prove That the Oxyds Which Are Formed on
the Surface of the Metallic Disks of the Galvanic Pile Do Not Impede the Effect
of Its Action, as Has Been Supposed; and That It
Has Even the Property of Supplying the Place of the Circles of Cloth Usually
Employed in the Pile of Volta, and Preserves Its Action
Fifteen or Twenty Days

**Published:** 1803-07-01

**Authors:** 


EXPERIMENTS, *which tend to prove that the* OXYDS *which are formed on the* SURFACE *of the* METALLIC DISKS *of the* GALVANIC PILE *do not impede the* EFFECT *of its* ACTION, *as has been supposed; and that it has even the Property of supplying the Place of the Circles of Cloth usually employed in the Pile of* VOLTA, *and preserves its Action Fifteen or Twenty Days. By* LA GRAVE, M.G.S.

VOLTA, Vassali, &c. had supposed that this oxyd destroyed the effect of the pile. It is true that the more the surfaces of the metals are exposed, the sooner the effect is felt, and the greater its force. The author composed piles, the disks of which were polished, and the force of which increased as its height. I have, says the author, been obliged, from the inconvenience which resulted, to confine myself to the use of half the number usually employed. I have, however, formed piles with disks which I had oxydated even to the obliteration of every metallic appearance, and which produced not only a sensation, but which served me in every Galvanic experiment. I differ from the ordinary manner of composing these piles only in the greater number of disks which I used; but what is singular is, that the effect was the reverse of what is in general experienced. The pile, well oxydated, produces its effect immediately, and as immediately loses its power; mercury, on the contrary, only acquired it after five or six hours, and retained it fifteen, twenty, or twenty-five days; whereas the pile, as it is generally constructed, affects only for three or four hours. With these same disks I formed piles without any interposition of cloths, and with the alternate disposition of zinc and copper; the effect was nearly the same. The author thinks that the oxyd supplied the place of the cloth. He concludes by observing, that whether this property arises from the power of the oxyds to retain water, he conceives it a fact well worth consideration, and likely to throw much light on the composition of the Galvanic pile.

